# Fabrication and Characteristics of PCL Membranes Containing Strontium-Substituted Hydroxyapatite Nanofibers for Guided Bone Regeneration

**DOI:** 10.3390/polym11111761

**Published:** 2019-10-27

**Authors:** Shiao-Wen Tsai, Wen-Xin Yu, Pai-An Hwang, Yu-Wei Hsu, Fu-Yin Hsu

**Affiliations:** 1 Graduate Institute of Biomedical Engineering, Chang Gung University, Taoyuan 303, Taiwan; swtsai@mail.cgu.edu.tw; 2 Department of Periodontics, Chang Gung Memorial Hospital, Taipei 105, Taiwan; 3 Department of Bioscience and Biotechnology, National Taiwan Ocean University, Keelung 202, Taiwan; andy54861@gmail.com (W.-X.Y.); amperehwang@gmail.com (P.-A.H.); qazwest74@gmail.com (Y.-W.H.)

**Keywords:** poly(ε-caprolactone), strontium, hydroxyapatite, guided bone regeneration

## Abstract

Poly(ε-caprolactone) (PCL) membranes have been widely used in guided tissue regeneration (GTR) and guided bone regeneration (GBR). In addition, hydroxyapatite is the major inorganic component and an essential composition of hard bone and teeth. Recently, numerous studies have demonstrated that strontium-substituted hydroxyapatite (SrHA) not only enhances osteogenesis but also inhibits adipogenesis of mesenchymal stem cells. Therefore, SrHA incorporated into PCL could be an alternative material for GBR. In this study, strontium-substituted hydroxyapatite nanofibers (SrHANFs) were fabricated by a sol–gel route followed by electrospinning. We then fabricated PCL–SrHANF membranes as cell culture substrates and assessed the cellular behavior of osteoblast-like cells. Based on the observations of alkaline phosphatase (ALP) activity, bone sialoprotein (BSP) and osteocalcin (OCN) immunofluorescence staining, and Alizarin Red-S staining of cells cultured on the PCL–SrHANF and PCL membranes, we concluded that SrHANFs can promote the differentiation and mineralization of osteoblast-like cells and that PCL–SrHANF membranes have potential for GBR applications.

## 1. Introduction

Membranes are mainly used for guided tissue regeneration (GTR) or guided bone regeneration (GBR) in the treatment of periodontitis. The basic requirements of GTR/GBR membranes include their being stretchable, highly biocompatible, and suitably biodegradable [1,2]. Polycaprolactone (PCL) is a semicrystalline hydrophobic biodegradable polyester and exhibits a slow degradation rate and strong mechanical strength. PCL was approved by the Food and Drug Administration as a medical and drug delivery device to be used in humans. Hence, PCL has attracted much attention in tissue engineering. However, the high hydrophobicity of PCL relative to that of natural extracellular matrix (ECM) leads to poor cell behavior [3]. Some studies have demonstrated that adding bioactive materials, such as hydroxyapatite (HAp), [4] tricalcium phosphate [5], and bioactive glass [6], to PCL can improve the hydrophilicity of PCL. Lee et al. showed that the incorporation of bioactive glass can significantly enhance cell activities on PCL membranes, indicating favorable osteoconductivity and osteoinductivity for GBR [7]. Shor et al. found that PCL–HA scaffolds had significantly higher compressive modulus than did PCL scaffolds. Moreover, they found that PCL–HA scaffolds had higher ALP activity and showed more mineralization of matrix than PCL scaffolds [8].

HAp is the main inorganic composition of tooth enamel, dentine, and bone. Synthetic HAp is widely used as a component of fillers in bones and teeth due to its excellent biocompatibility, bioactivity, and osteoconductivity. Strontium (Sr) is a divalent cation that can partially substitute Ca^2+^ in the crystal lattice of HAp. Zhang et al. found that strontium-substituted hydroxyapatite (SrHAp) has higher solubility than does pure HAp due to a difference in ionic radius, which contributes to perturbations in the crystal lattice [9]. Furthermore, Tsai et al. noted that the substitution of Ca with Sr leads to increased d-spacings, causing the dissolution ratio of SrHAp to increase with the dose of Sr [10].

Previous studies have demonstrated the osteogenic effect of SrHAp on osteoblasts [11,12,13,14,15]. In our past research, strontium-substituted hydroxyapatite nanofibers (SrHANFs) were fabricated and demonstrated to possess excellent drug-loading efficiency and the ability to release tetracycline in a sustained manner, leading to the maintenance of antibacterial activity for over 3 weeks [10]. However, the effect of SrHANFs on the osteogenic differentiation of osteoblast cells has not been evaluated.

Therefore, the aim of this work was to fabricate a bioactive and biodegradable composite membrane containing PCL and SrHANFs for GBR applications and to assess the cellular behavior of osteoblast-like cells on PCL–SrHANF membranes.

## 2. Materials and Methods

### 2.1. Reagents

Triethyl phosphite (TEP) was purchased from Merck (Darmstadt, Germany). Poly(ethylene glycol)-poly(propylene glycol)-poly(ethylene glycol) (Pluronic P123, *M*_W_ 5,800), poly(vinyl pyrrolidone) (PVP, *M*_W_ 40,000), PCL (*M*_W_ 70,000), calcium nitrate tetrahydrate, strontium nitrate, and cetyltrimethylammonium bromide (CTAB) were purchased from Sigma-Aldrich Chemical Company (St. Louis, MO, USA). All other chemicals used were reagent grade, unless otherwise stated.

### 2.2. Synthesis and Characterization of SrHANFs

The SrHANFs were synthesized based on our previously reported method [10], and the molar ratio of Sr/(Sr + Ca) was 30 mol%, and the (Sr + Ca)/P molar ratio was 1.67. Briefly, 0.5 g of CTAB and 3.086 mL of TEP were mixed in 5 mL of an ethanol aqueous solution (50%, *v*/*v*) and continuously stirred until a stable and clear solution was obtained. A total of 4.896 g of calcium nitrate tetrahydrate was dissolved in 5 mL of an ethanol solution (95%, *v*/*v*), and 1.876 g of strontium nitrate was dissolved in 3 mL of deionized water. Subsequently, Ca(NO_3_)_2_ and Sr(NO_3_)_2_ solutions were slowly added into the above TEP/CTAB solution to form a precursor solution.

Next, 0.585 g of PVP and 2.25 g of P123 were dissolved in 7 mL of absolute ethanol and incorporated into 3 ml of the above precursor solution. The mixture solution was placed into an oven at 60 °C for 12 h. Subsequently, the mixture solution was taken up in a syringe fitted with a needle (18 G) and operated under a steady flow rate (1.27 mL/h) and electrical field (1.3 kV/cm, Spellman SL 60^®^, New York, NY, USA). The mixture solution was ejected, and the formed nanofibers were collected on an aluminum substrate and then calcined at 800 °C under a nitrogen atmosphere to obtain the SrHANFs.

### 2.3. Characterization of the SrHANFs

The morphology of the SrHANFs was observed using a scanning electron microscope (SEM, ZEISS Sigma, Dresden, Germany) that operated at an accelerating voltage of 15 kV. The average diameter of the SrHANFs was determined from the SEM images using image analysis software (Image-Pro Express software, Media Cybernetics, Rockville, MD, USA). The pore structure of the SrHANFs was observed using transmission electron microscopy (TEM, JEOL JEM-2100, Tokyo, Japan) at an accelerating voltage of 100 kV.

The phase of the SrHANFs was characterized by a Bruker D2-Phaser powder diffractometer (Madison, WI, USA) equipped with copper K_α_1 radiation (λ = 1.5406 Å). The X-ray diffraction patterns of the SrHANFs were recorded in the angular range (2θ) from 20° to 60° with a step size of 0.04°.

### 2.4. Preparation of PCL and PCL–SrHANF Membranes

The nonwoven structure of the SrHANFs was reduced to fragments by a sonicator. The PCL and PCL–SrHANF membranes were fabricated by the solvent casting technique. First, PCL (130 mg) was dissolved in acetone (1 mL), and varying amounts of SrHANF fragments (6.5, 13, and 19.5 mg) were added later. The mixture solution was poured into a glass dish and dried to form PCL and PCL–SrHANF membranes. The sample notations and amounts of PCL and SrHANFs used for the fabrication of PCL–SrHANF membranes are shown in Table 1.

### 2.5. Characterization of PCL–SrHANF Membranes

The morphology of the PCL–SrHANF membranes was observed by SEM. Briefly, the membranes were sputter-coated with gold and were visualized by SEM at an accelerating voltage of 10 kV. The chemical structure of the PCL–SrHANF membranes was analyzed through Fourier-transform infrared (FTIR) spectroscopy over the range of 4000–400 cm^−1^ at a resolution of 4 cm^−1^. X-ray diffraction (XRD) tests of the PCL–SrHANF membranes were performed on a Bruker D2-Phaser diffractometer (Bruker, Madison, WI, USA) in the angular range (2θ) from 20° to 60° with a step size of 0.04°.

### 2.6. Cellular Adhesion and Proliferation on PCL and PCL–SrHANF Membranes

The PCL and PCL–SrHANF membranes (1 × 1 cm^2^) were sterilized by overnight exposure to UV light prior to cell culture. The sterilized membranes were placed into 24-well tissue culture plates containing a suspension of MG63 osteoblast-like cells (BCRC NO. 60279) (1 × 10^4^ cells/well) in minimum essential medium (MEM) supplemented with 10% (*v*/*v*) fetal bovine serum (FBS), 50 µg/mL ascorbic acid, 10 mM ß-glycerophosphate, 100 U/mL penicillin, and 100 µg/mL streptomycin. Cell attachment was assayed at 1, 2, and 4 h, and cell proliferation was assayed on days 1, 3, and 7 using the 3-(4,5-dimethylthiazol-2-yl)-2,5-diphenyltetrazolium bromide (MTT) assay.

### 2.7. Cytoskeleton Organization and Immunofluorescence

MG63 osteoblast-like cells cultured on the various membranes were stained with rhodamine-phalloidin to observe the cytoskeletal organization and stained with osteoblast-specific marker proteins, such as osteocalcin (OCN) and bone sialoprotein (BSP), to evaluate differentiation. The membranes were first fixed with 3.7% paraformaldehyde in 0.02 M phosphate-buffered saline (PBS, pH = 7.4). Subsequently, the membranes were rinsed in PBS with 0.1% Triton X-100 for 5 min and were blocked in PBS with 1% bovine serum albumin for 1 h to reduce nonspecific background staining. After blocking, the membranes were incubated with Alexa Fluor® 488 phalloidin or a primary antibody against osteoblast-specific marker protein OCN (AB10911, Millipore, Temecula, CA, USA) or BSP (AB1854, Millipore, Temecula, CA, USA). Subsequently, the membranes were incubated with a secondary antibody (rhodamine-conjugated goat antirabbit antibody, sc2091, Santa Cruz, CA, USA). Next, the membranes were incubated in PBS with 0.1% 4’,6-diamidino-2-phenylindole (DAPI) to stain the cellular nucleus. Finally, the membranes were washed with PBS and observed under a laser scanning confocal microscope (LSCM, Zeiss LSM 780 META, Carl Zeiss Inc., Oberkochen, Germany). The cytoskeleton, osteoblast-specific marker proteins, and nuclei were stained green, red, and blue, respectively.

### 2.8. Alkaline Phosphatase (ALP) Activity of MG63 Osteoblast-Like Cells on Membranes

The cell-seeded membranes were washed with PBS and suspended in lysis solution (0.1 M glycine, 1 mM MgCl_2_, and 1% Triton X-100 in PBS) for 20 min. After lysis, 50 μL of the lysate was incubated with 150 μL of the ALP activity reagent (Randox® ALP detection kit, UK) for 1 h at 37 °C. The absorbance of the solution was determined by monitoring the light absorbance at 405 nm. The ALP activity (OD 405 nm) was normalized by the absorbance of MTT (OD 590 nm).

### 2.9. Alizarin Red-S (ARS) Staining for Mineralization

The formation of calcium phosphate by the MG63 osteoblast-like cells was determined using the ARS assay [16]. The medium was removed, and the membranes were washed with PBS and fixed in 3.7% (*v*/*v*) formaldehyde at room temperature for 15 min. The membranes were washed with deionized water and then stained with a 2% ARS solution (pH 4.2) for 15 min. Finally, the membranes were washed several times with deionized water to remove the remaining stain. Calcium deposits can be observed by the red color under microscopy (Nikon TS-100, Tokyo, Japan). The bound ARS was dissolved in 10% acetic acid to quantify the staining. [17] The concentration of ARS was determined by absorbance measurement at 430 nm wavelength on a multiplate ELISA reader. To account for the interference of the binding of ARS to SrHANFs during mineralization, PCL or PCL–SrHANF membranes without MG63 cell seeding were used as controls. The ARS concentration of the control membranes was subtracted from the ARS concentration of the corresponding membranes with MG63 osteoblast-like cells to yield the net mineral concentration synthesized by the MG63 osteoblast-like cells.

### 2.10. Statistical Analyses

Statistical analyses were performed using SPSS v.10. Cellular attachment and proliferation, ALP activity and mineralization were analyzed by the nonparametric Mann–Whitney U test. Differences at *p* < 0.05 were considered statistically significant.

## 3. Results and Discussion

### 3.1. Characterization of SrHANFs

**Figure 1:**
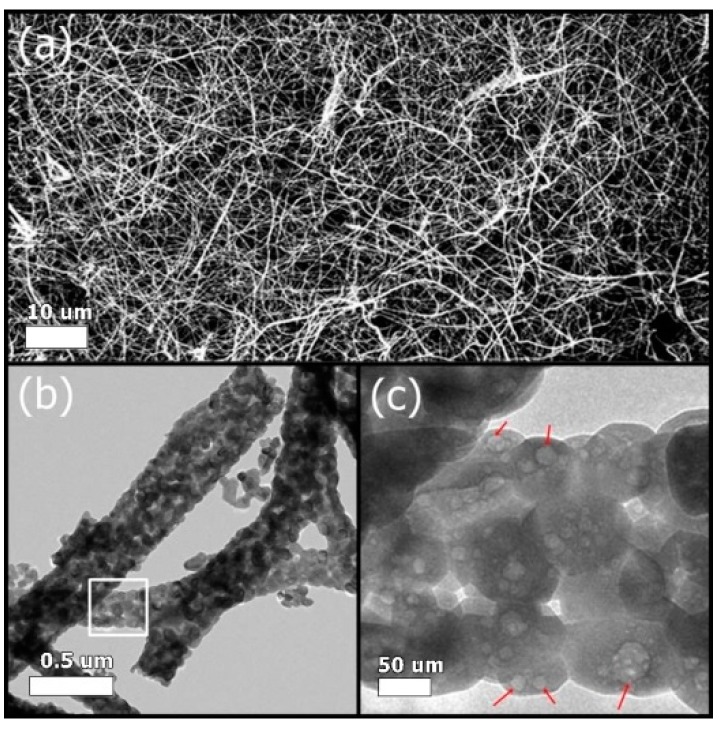
(**a**) SEM and (**b**) TEM images of the SrHANFs. (**c**) An enlarged graph in the white square of (**b**). The red arrows in (**c**) indicate mesopores within the nanocrystals.

The morphology and microstructure of the SrHANFs were observed under SEM and TEM. Figure 1a shows an SEM image of the SrHANFs. The average diameter of SrHANF was 291 ± 133 nm. The (Ca+Sr)/P ratio of SrHANF from ICP-OES analysis was approximately 1.68, which was very close to the stoichiometric HAp ratio (Ca/P = 1.67). The TEM image of the SrHANFs shows that the nanofibers were composed of a number of nanocrystals and reveals the existence of mesopores within the nanocrystal (shown in Figure 1b,c).

### 3.2. Characterization of PCL–SrHANF Membranes

**Figure 2:**
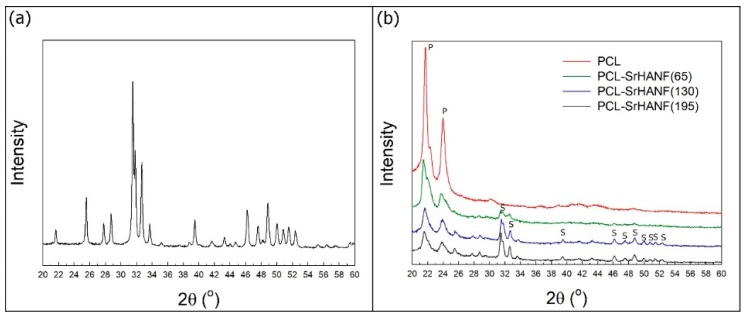
XRD patterns of the (**a**) SrHANFs and (**b**) PCL–SrHANF membranes. P: PCL, S: SrHA.

Figure 2 shows the wide-angle XRD patterns of the SrHANF, PCL, and PCL–SrHANF membranes. As shown in Figure 2a, the main phase of the SrHANFs was Sr-substituted HAp (PDF card: #89-5631). PCL is a semicrystalline polymer with diffraction peaks at approximately 21.5° and 23.9°, which are assigned to the (110) and (200) planes, respectively [18]. XRD patterns of the PCL–SrHANF membranes show the presence of two phases, PCL and SrHANFs (Figure 2b). The broad amorphous halos increased with increasing content of the SrHANF component. This result suggested that during crystallization, the SrHANFs are occluded in intercrystalline domains, hindering the crystallization of PCL [19].

**Figure 3:**
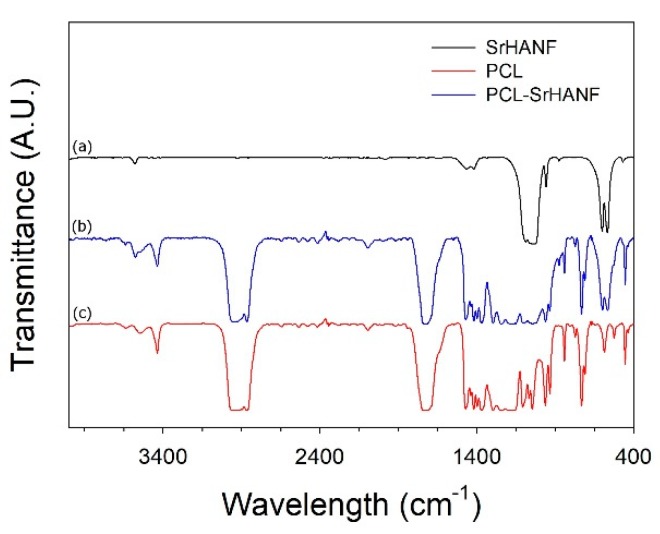
FTIR spectra of (**a**) SrHANFs, (**b**) PCL–SrHANF, and (**c**) PCL.

The FTIR spectra of the PCL, SrHANFs, and PCL–SrHANF are shown in Figure 3. The characteristic bands of PCL were observed at approximately 1400 (C–O stretching), 1750 (C=O stretching), and 2900 cm^−1^ (C–H stretching). The characteristic bands of the SrHANFs were observed at approximately 566 and 609 cm^−1^ (P–O bending) and at 960 cm^−1^ and 1000~1100 cm^−1^ (P–O stretching) in PO_4_^3−^. The corresponding bands of SrHANFs and PCL in the PCL–SrHANF spectrum confirmed both compounds in the PCL–SrHANF.

**Figure 4:**
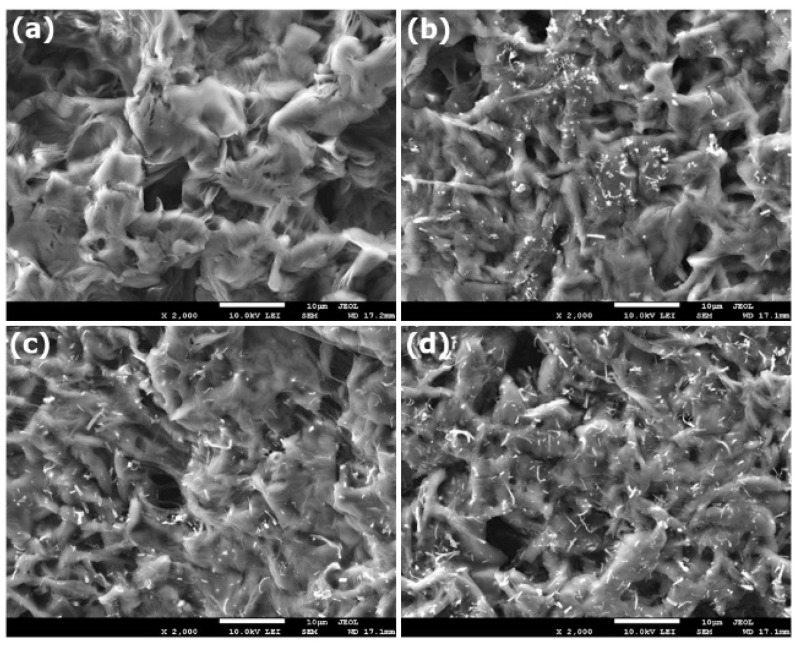
SEM images of (**a**) PCL, (**b**) PCL–SrHANF (65), (**c**) PCL–SrHANF (130), and (**d**) PCL–SrHANF (195). Scale bar = 10 μm.

Figure 4 presents the surface morphology of PCL and PCL–SrHANF membranes containing SrHANFs at percentages of 6.5% to 19.5%. Many pores were present on the PCL and PCL–SrHANF membrane surfaces. Moreover, the SrHANF fragments dispersed into the PCL substrate.

### 3.3. Cellular Behavior on the Membranes

**Figure 5:**
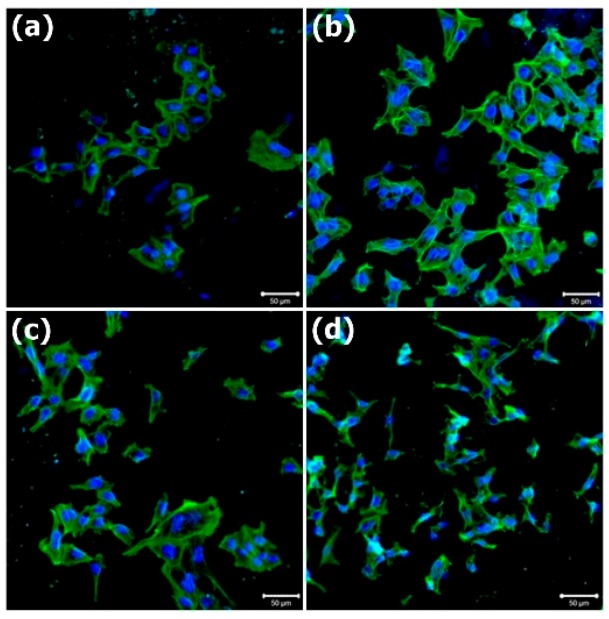
Representative images of MG63 osteoblast-like cells cultured on (**a**) PCL, (**b**) PCL–SrHANF(65), (**c**) PCL–SrHANF (130), and (**d**) PCL–SrHANF(195) after 1 day of culture. Cytoskeletal F-actin is stained green with fluorescein isothiocyanate (FITC), and cell nuclei are stained blue with 4’,6-diamidino-2-phenylindole (DAPI). Scale bar = 50 μm.

The MG63 osteoblast-like cells adhering to the PCL and PCL–SrHANF membranes were stained with fluorescein isothiocyanate (FITC)–phalloidin for detecting the actin cytoskeleton and with DAPI for detecting nuclei and were observed under LSCM (as shown in Figure 5). The cells on the PCL membrane were round (Figure 5a), whereas the cells on the PCL–SrHANF membranes gradually adopted a bipolar shape as the amount of SrHANFs increased (Figure 5b–d). Webb noted that hydrophilic surfaces are better than hydrophobic surfaces for cell spreading [20].

**Figure 6:**
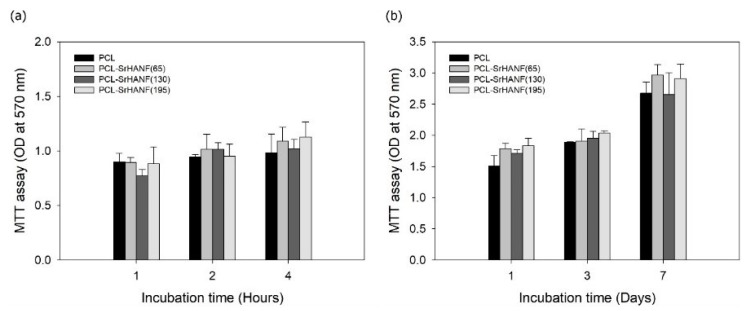
3-(4,5-dimethylthiazol-2-yl)-2,5-diphenyltetrazolium bromide (MTT) assay quantifying cell attachment and proliferation on PCL–SrHANF membranes. (**a**) Attached MG63 osteoblast-like cells on various membranes after culturing for up to 4 h. (**b**) Viability of MG63 osteoblast-like cells on various membranes after culturing for up to 7 days. The data are presented as the mean ± SD, n = 3.

**Figure 7:**
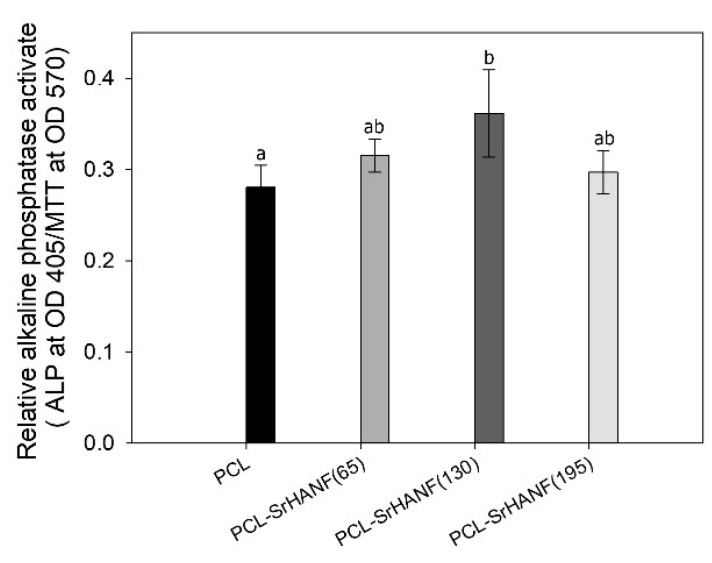
Relative alkaline phosphatase (ALP) activity of MG63 osteoblast-like cells on PCL–SrHANF membranes after 7 days of incubation. The data are presented as the mean ± SD, n = 3. Different letters represent significance at *p* < 0.05.

In addition to influencing hydrophilicity, the molecular composition of the matrix can affect cell behavior, such as attachment, growth, proliferation, and differentiation [21,22]. The effect of SrHANF content in the PCL–SrHANF membranes on cell attachment, proliferation, and differentiation was examined by MTT assay and analysis of ALP activity, as shown in Figure 6 and Figure 7, respectively. After 4 h of cell culture, the membrane with 19.5 wt % SrHANFs showed the highest level of cell attachment, but there were no significant differences in attachment level between this membrane and the other membranes (*p* > 0.05). In addition, the PCL–SrHANF membranes showed higher cell proliferation than did the pure PCL membrane after 7 days of cell culture. However, no statistically significant differences were observed between the PCL and PCL–SrHANF membranes.

Xue et al. demonstrated that SrHA could enhance the proliferation of osteoblast cells [23]. Caverzasio noted that the presence and release of strontium ions could enhance cell proliferation [24]. However, the SrHANFs were not observed to enhance osteoblastic proliferation in the present study. Shu et al. found that HAp could suppress the growth of osteoblastic cells. Tsai et al. [25] reported that the dissolution of HAp particles causes a high local calcium ion concentration that could stimulate osteoblasts to immediately switch from the proliferation stage to the differentiation stage. Lion et al. [26] found that the proliferation of bone marrow stromal cells on PCL–PDIPF (polydiisopropyl fumarate) film containing 5% strontium was significantly decreased relative to that of PCL–PDIPF film containing 1% strontium and pure PCL–PDIPF film. Our previous study [10] found that the dissolution rate of SrHANFs increased as the doping amount of Sr increased due to the difference in ionic radius which perturbed the crystal lattice. The concentrations of Ca^2+^ and Sr^2+^ released from 10 mg SrHANFs with a Sr/(Sr + Ca) molar ratios of 30% to 1 ml de-ionized water after 1 day were approximately 0.85 mM and 0.37 mM, respectively. Hence, we speculate that the dissolution of SrHANFs causes the concentrations of calcium and strontium ions to increase, thus affecting cell proliferation.

The osteoblastic differentiation progresses through three stages: proliferation with the secretion of ECM, ECM maturation, and ECM mineralization. To evaluate the osteogenic differentiation of MG63 osteoblast-like cells on the PCL and PCL–SrHANF membranes, the ALP activity and the presence of OCN and BSP were examined. ALP activity is an early marker of the osteoblastic phenotype. The MG63 osteoblast-like cells cultured on the PCL and PCL–SrHANF membranes were evaluated by assaying their ALP activity after culturing for 7 days. The PCL–SrHANF membranes showed higher ALP activity than that of the PCL membranes (shown as Figure 7) because the dissolved SrHANFs on PCL cause high local calcium ion and strontium ion concentrations, promoting the differentiation of osteoblasts. However, the ALP activity of MG63 osteoblast-like cells on PCL–SrHANF (195) was lower than that on PCL–SrHANF (130) after 7 days of cell culture. ALP activity is upregulated at the onset of cell differentiation but subsequently decreases as cell differentiation progresses [27]. The amount of SrHANF on PCL–SrHANF(195) was larger than that on PCL–SrHANF (130). This result indicates that the onset of cell differentiation on PCL–SrHANF(195) occurs earlier than that on PCL–SrHANF (130).

**Figure 8:**
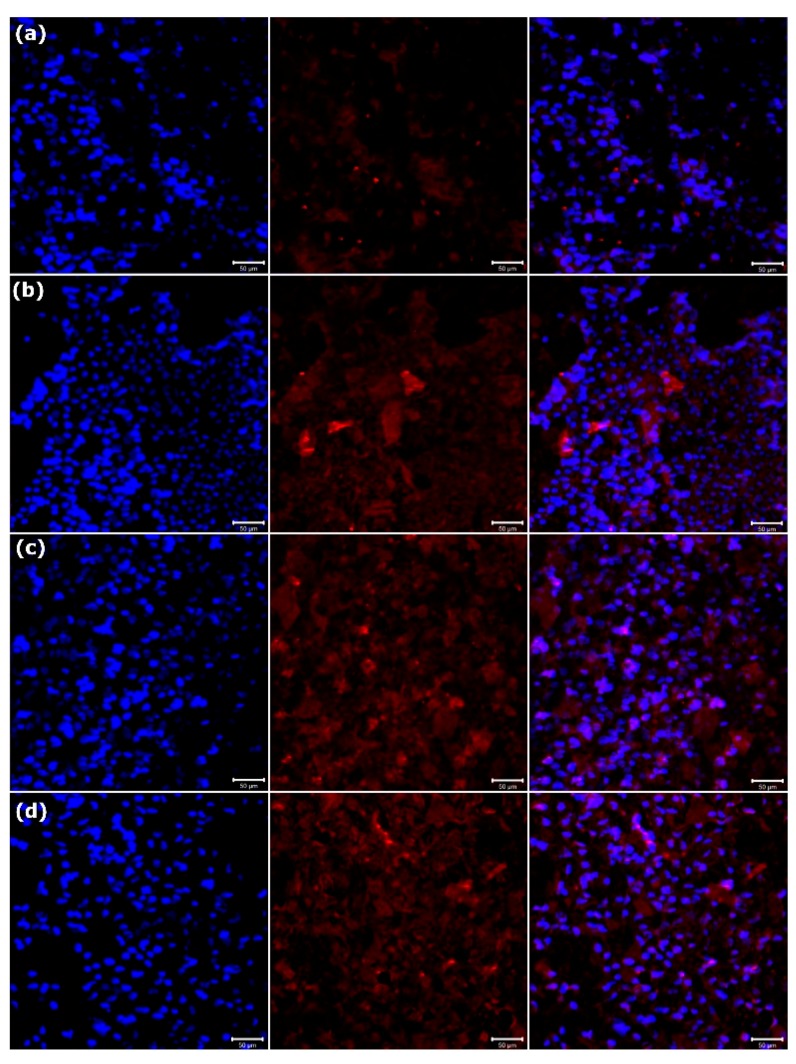
Immunofluorescence analysis of bone sialoprotein (BSP) on (**a**) PCL, (**b**) PCL–SrHANF (65), (**c**) PCL–SrHANF (130), and (**d**) PCL–SrHANF (195) after 14 days of culture. The MG63 osteoblast-like cells were stained with BSP (**red**), and the cell nuclei were stained with DAPI (**blue**). Scale bar = 50 μm.

**Figure 9:**
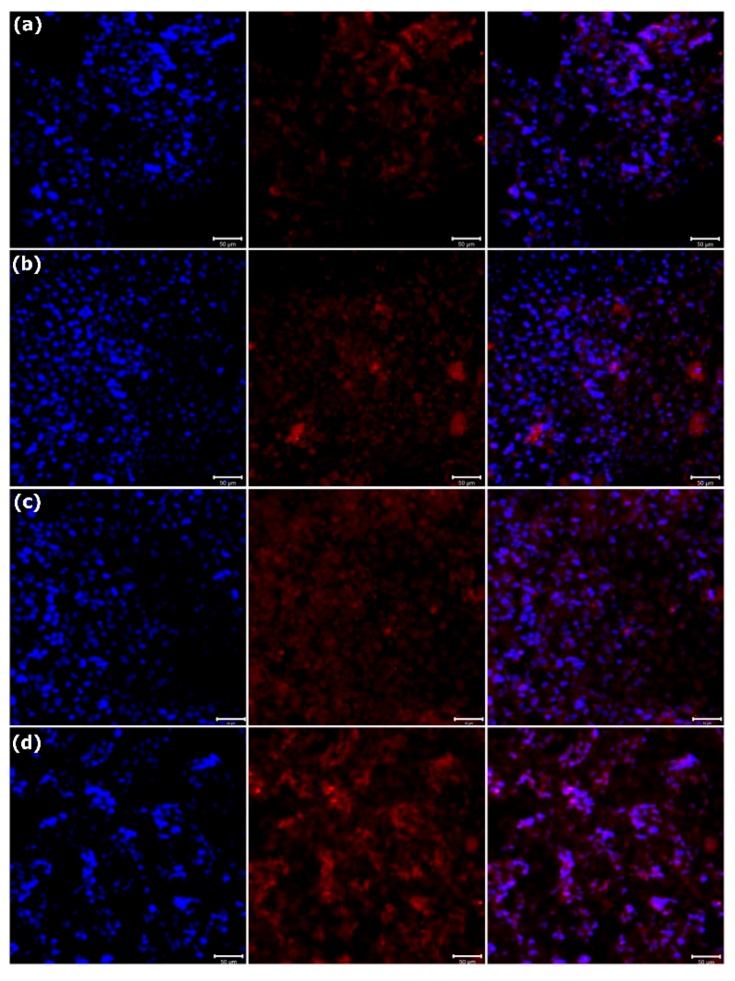
Immunofluorescence analysis of OCN on (**a**) PCL, (**b**) PCL–SrHANF (65), (**c**) PCL–SrHANF (130), and (**d**) PCL–SrHANF (195) after 14 days of culture. The MG63 osteoblast-like cells were stained with OCN (**red**), and the cell nuclei were stained with DAPI (**blue**). Scale bar = 50 μm.

BSP is a phosphorylated protein in the bone matrix and has been proposed to initiate mineralization of the extracellular matrix by participating in HAp nucleation [28]. OCN is a bone-specific protein synthesized by osteoblasts during the matrix mineralization stage [29]. Hence, BSP and OCN are regarded as markers of osteoblasts at their middle and mature stages of differentiation, respectively. The expression of BSP and OCN proteins on the PCL and PCL–SrHANF membranes was evaluated by immunofluorescence staining of the MG63 osteoblast-like cells. Compared with that in cells cultured on the PCL membranes, the expression of BSP and OCN proteins in cells cultured on the PCL–SrHANF membranes was significantly higher on day 14 (shown as Figure 8 and Figure 9). Gentlemen et al. [30] found the increased amount of Sr ions released from PCL-Sr doping bioglass scaffolds promoted osteoblast differentiation over that observed on PCL scaffolds alone. In addition, Ren et al. [31] found significantly higher OCN gene expression in PCL–Sr doping bioglass scaffolds than on PCL after 14 days of culture. Hence, we speculate that the Sr^2+^ ions released from the PCL–SrHANF membranes enhance the expression and activity of osteogenesis-related genes and proteins through the interaction of calcium-sensing receptor (CaR) with cells to activate inositol-1,4,5-triphosphate production and mitogen-activated protein kinase (MAPK) signaling, which regulate osteogenic differentiation [24].

**Figure 10:**
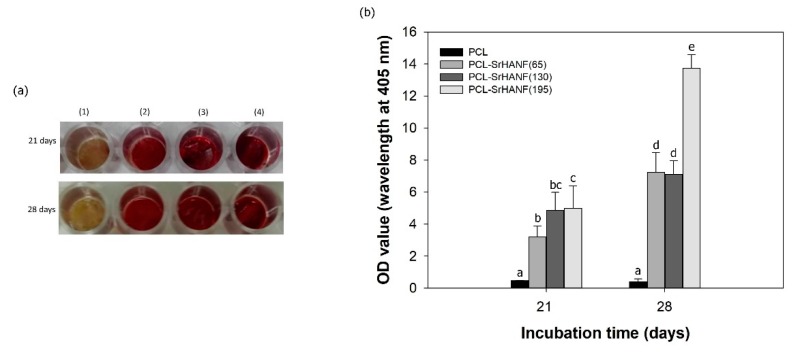
Effects of SrHANF on bone mineralization in MG63 osteoblast-like cells. (**a**) Optical images and (**b**) quantification of mineral deposition by ARS staining after 21 and 28 days of incubation. (1) PCL, (2) PCL–SrHANF (65), (3) PCL–SrHANF (130), and (4) PCL–SrHANF (195). The data represent the mean ± SD, *n* = 3. Different letters represent significance at *p* < 0.05.

The mineralization of MG63 osteoblast-like cells on the PCL and PCL–SrHANF membranes was quantitatively determined by colorimetric calcium quantification (Figure 10). The mineralization process was time-dependent, and a significantly higher level of mineralization in the MG63 osteoblast-like cells was observed on PCL–SrHANF membranes than on PCL on day 21 and day 28. In addition, increasing the amount of SrHANFs on the PCL–SrHANF membranes increases the level of mineralization of MG63 osteoblast-like cells on membranes. These results indicate that the presence of SrHANFs in the PCL membranes promotes osteoblast differentiation and activity.

## 4. Conclusions

An organic–inorganic composite membrane for guided bone regeneration was fabricated for the first time by incorporating fragments of strontium-substituted hydroxyapatite nanofiber into PCL. Analyses of ALP activity and protein expression of OCN and BSP indicated that the PCL–SrHANF composite membrane possessed elevated osteogenic potential relative to that of PCL membrane alone. The findings indicate that PCL–SrHANF membranes are more bioactive than PCL membranes and could potentially be used as a GBR membrane.

## Figures and Tables

**Table 1:** Sample notations and amounts of poly(ε-caprolactone) (PCL) and strontium-substituted hydroxyapatite nanofibers (SrHANFs) for the fabrication of PCL–SrHANF membranes.

Sample Notation	PCL (mg/mL)	SrHANF (mg/mL)
PCL	130	0
PCL–SrHANF(65)	130	6.5
PCL–SrHANF(130)	130	13.0
PCL–SrHANF(195)	130	19.5
